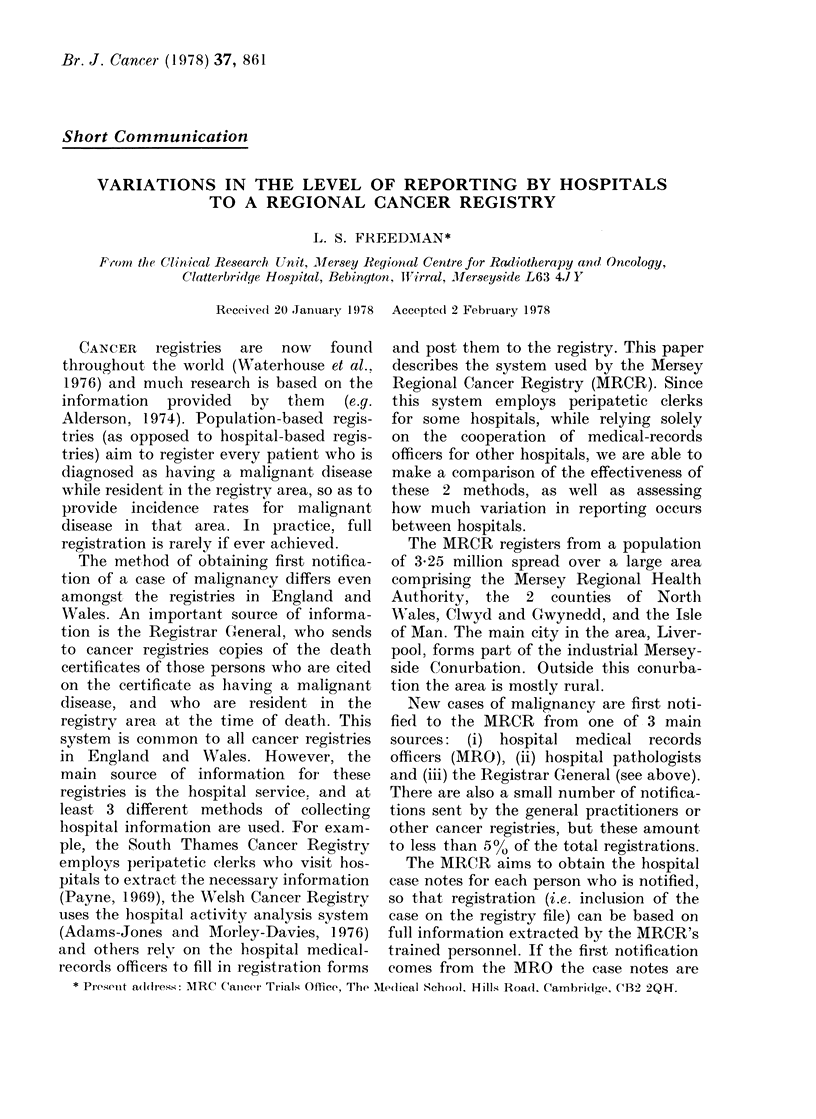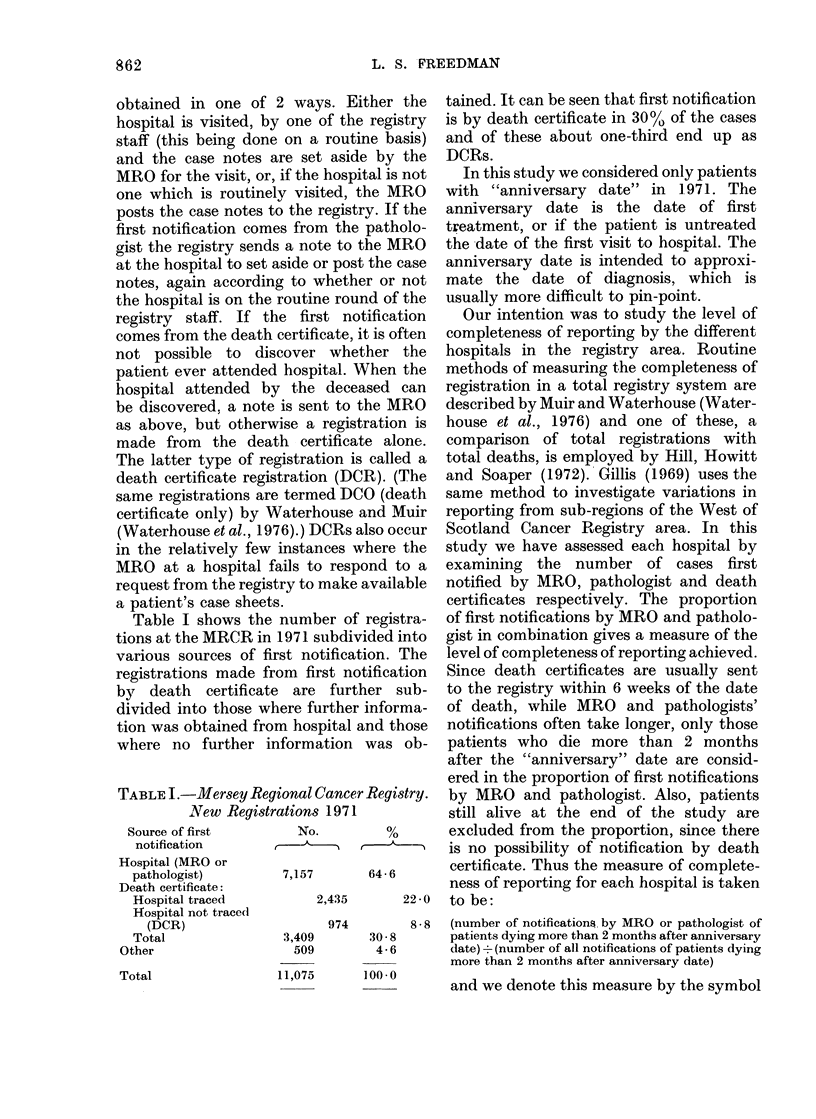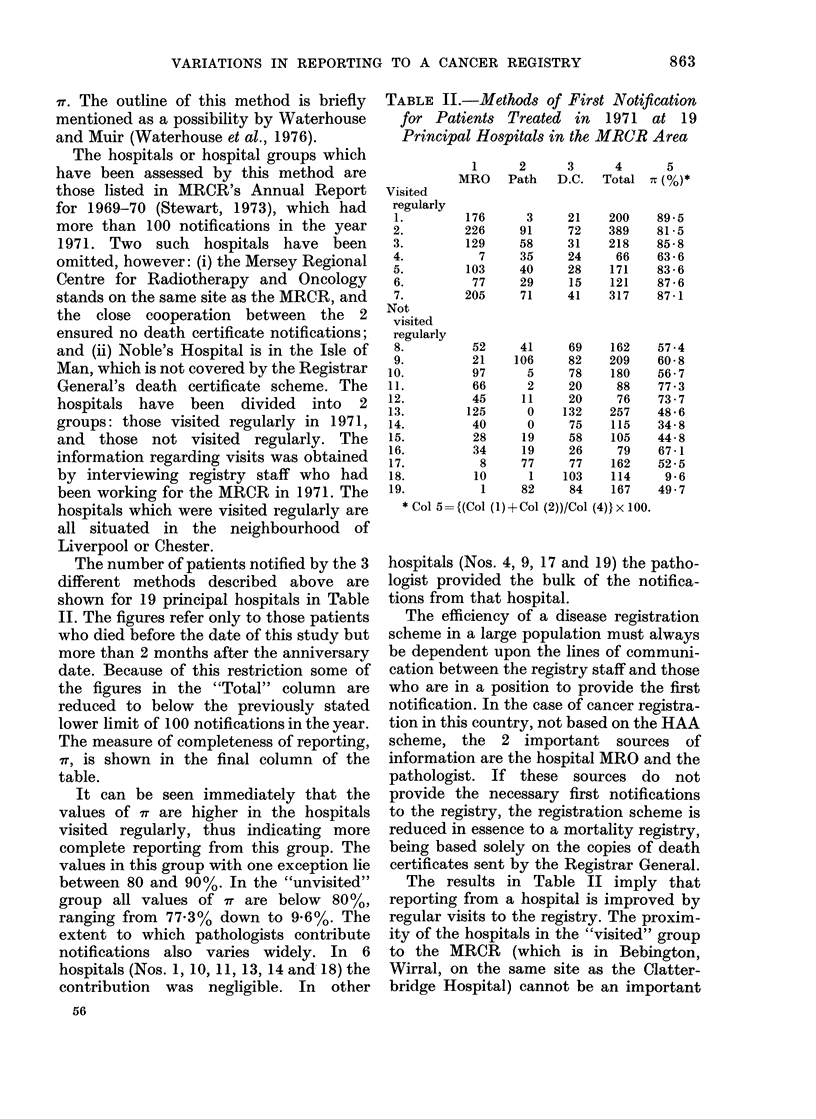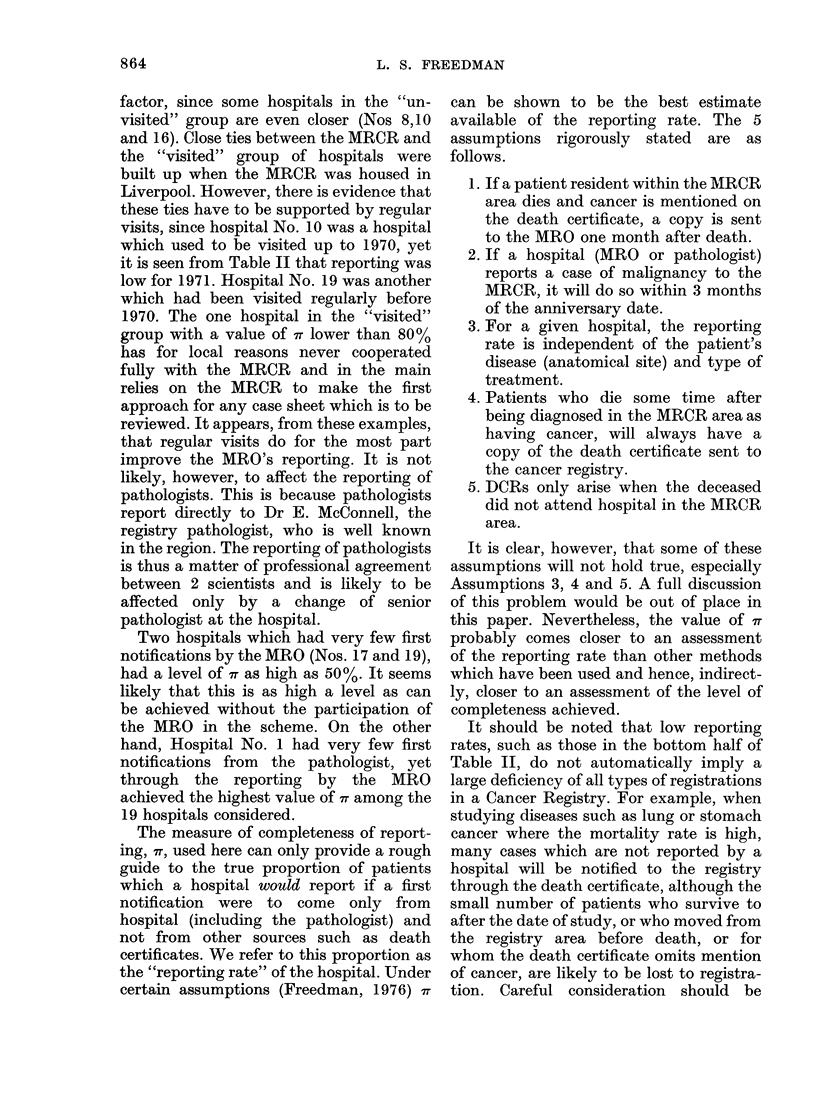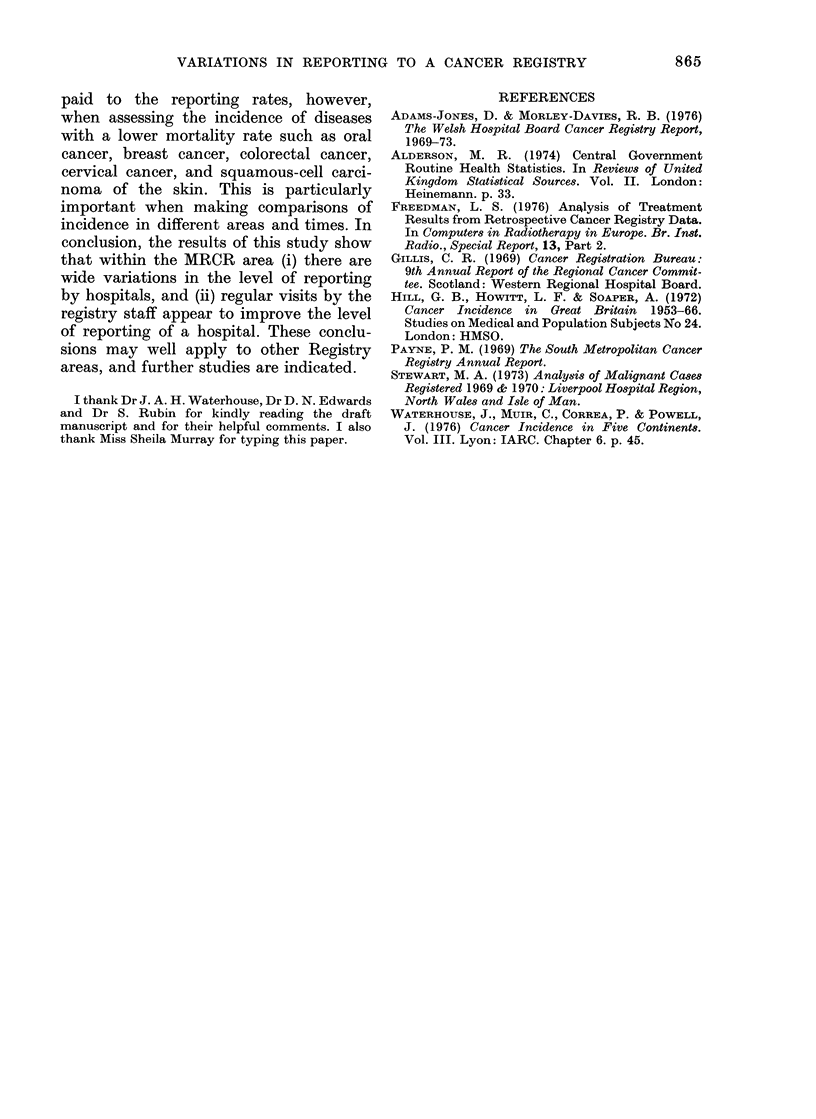# Variations in the level of reporting by hospitals to a regional cancer registry.

**DOI:** 10.1038/bjc.1978.126

**Published:** 1978-05

**Authors:** L. S. Freedman


*Br. J. Cancer* (1978) **37**, 861

***Short Communication***

### VARIATIONS IN THE LEVEL OF REPORTING BY HOSPITALS TO A REGIONAL CANCER REGISTRY

L. S. FREEDMAN*

*From the Clinical Research Unit, Mersey Regional Centre for Radiotherapy and Oncology, Clatterbridge Hospital, Bebington, Wirral, Merseyside L63 4JY*

Received 20 January 1978 Accepted 2 February 1978

CANCER registries are now found throughout the world (Waterhouse *et al.*, 1976) and much research is based on the information provided by them (*e.g.* Alderson, 1974). Population-based registries (as opposed to hospital-based registries) aim to register every patient who is diagnosed as having a malignant disease while resident in the registry area, so as to provide incidence rates for malignant disease in that area. In practice, full registration is rarely if ever achieved.

The method of obtaining first notification of a case of malignancy differs even amongst the registries in England and Wales. An important source of information is the Registrar General, who sends to cancer registries copies of the death certificates of those persons who are cited on the certificate as having a malignant disease, and who are resident in the registry area at the time of death. This system is common to all cancer registries in England and Wales. However, the main source of information for these registries is the hospital service, and at least 3 different methods of collecting hospital information are used. For example, the South Thames Cancer Registry employs peripatetic clerks who visit hospitals to extract the necessary information (Payne, 1969), the Welsh Cancer Registry uses the hospital activity analysis system (Adams-Jones and Morley-Davies, 1976) and others rely on the hospital medical-records officers to fill in registration forms and post them to the registry. This paper describes the system used by the Mersey Regional Cancer Registry (MRCR). Since this system employs peripatetic clerks for some hospitals, while relying solely on the cooperation of medical-records officers for other hospitals, we are able to make a comparison of the effectiveness of these 2 methods, as well as assessing how much variation in reporting occurs between hospitals.

The MRCR registers from a population of 3·25 million spread over a large area comprising the Mersey Regional Health Authority, the 2 counties of North Wales, Clwyd and Gwynedd, and the Isle of Man. The main city in the area, Liverpool, forms part of the industrial Merseyside Conurbation. Outside this conurbation the area is mostly rural.

New cases of malignancy are first notified to the MRCR from one of 3 main sources: (i) hospital medical records officers (MRO), (ii) hospital pathologists and (iii) the Registrar General (see above). There are also a small number of notifications sent by the general practitioners or other cancer registries, but these amount to less than 5% of the total registrations.

The MRCR aims to obtain the hospital case notes for each person who is notified, so that registration (*i.e.* inclusion of the case on the registry file) can be based on full information extracted by the MRCR's trained personnel. If the first notification comes from the MRO the case notes are

* Present address: MRC Cancer Trials Office, The Medical School, Hills Road, Cambridge, CB2 2QH.

obtained in one of 2 ways. Either the hospital is visited, by one of the registry staff (this being done on a routine basis) and the case notes are set aside by the MRO for the visit, or, if the hospital is not one which is routinely visited, the MRO posts the case notes to the registry. If the first notification comes from the pathologist the registry sends a note to the MRO at the hospital to set aside or post the case notes, again according to whether or not the hospital is on the routine round of the registry staff. If the first notification comes from the death certificate, it is often not possible to discover whether the patient ever attended hospital. When the hospital attended by the deceased can be discovered, a note is sent to the MRO as above, but otherwise a registration is made from the death certificate alone. The latter type of registration is called a death certificate registration (DCR). (The same registrations are termed DCO (death certificate only) by Waterhouse and Muir (Waterhouse *et al.*, 1976).) DCRs also occur in the relatively few instances where the MRO at a hospital fails to respond to a request from the registry to make available a patient's case sheets.

Table I shows the number of registrations at the MRCR in 1971 subdivided into various sources of first notification. The registrations made from first notification by death certificate are further subdivided into those where further information was obtained from hospital and those where no further information was obtained. It can be seen that first notification is by death certificate in 30% of the cases and of these about one-third end up as DCRs.

TABLE I.—*Mersey Regional Cancer Registry. New Registrations* 1971

| Source of first notification | No. | | % | |
|---|---|---|---|---|
| Hospital (MRO or pathologist) | 7,157 | | 64·6 | |
| Death certificate: | | | | |
| Hospital traced | | 2,435 | | 22·0 |
| Hospital not traced (DCR) | | 974 | | 8·8 |
| Total | 3,409 | | 30·8 | |
| Other | 509 | | 4·6 | |
| Total | 11,075 | | 100·0 | |

In this study we considered only patients with "anniversary date" in 1971. The anniversary date is the date of first treatment, or if the patient is untreated the date of the first visit to hospital. The anniversary date is intended to approximate the date of diagnosis, which is usually more difficult to pin-point.

Our intention was to study the level of completeness of reporting by the different hospitals in the registry area. Routine methods of measuring the completeness of registration in a total registry system are described by Muir and Waterhouse (Waterhouse *et al.*, 1976) and one of these, a comparison of total registrations with total deaths, is employed by Hill, Howitt and Soaper (1972). Gillis (1969) uses the same method to investigate variations in reporting from sub-regions of the West of Scotland Cancer Registry area. In this study we have assessed each hospital by examining the number of cases first notified by MRO, pathologist and death certificates respectively. The proportion of first notifications by MRO and pathologist in combination gives a measure of the level of completeness of reporting achieved. Since death certificates are usually sent to the registry within 6 weeks of the date of death, while MRO and pathologists' notifications often take longer, only those patients who die more than 2 months after the "anniversary" date are considered in the proportion of first notifications by MRO and pathologist. Also, patients still alive at the end of the study are excluded from the proportion, since there is no possibility of notification by death certificate. Thus the measure of completeness of reporting for each hospital is taken to be:

(number of notifications by MRO or pathologist of patients dying more than 2 months after anniversary date) ÷ (number of all notifications of patients dying more than 2 months after anniversary date)

and we denote this measure by the symbol

$\pi$. The outline of this method is briefly mentioned as a possibility by Waterhouse and Muir (Waterhouse *et al.*, 1976).

The hospitals or hospital groups which have been assessed by this method are those listed in MRCR's Annual Report for 1969–70 (Stewart, 1973), which had more than 100 notifications in the year 1971. Two such hospitals have been omitted, however: (i) the Mersey Regional Centre for Radiotherapy and Oncology stands on the same site as the MRCR, and the close cooperation between the 2 ensured no death certificate notifications; and (ii) Noble's Hospital is in the Isle of Man, which is not covered by the Registrar General's death certificate scheme. The hospitals have been divided into 2 groups: those visited regularly in 1971, and those not visited regularly. The information regarding visits was obtained by interviewing registry staff who had been working for the MRCR in 1971. The hospitals which were visited regularly are all situated in the neighbourhood of Liverpool or Chester.

The number of patients notified by the 3 different methods described above are shown for 19 principal hospitals in Table II. The figures refer only to those patients who died before the date of this study but more than 2 months after the anniversary date. Because of this restriction some of the figures in the "Total" column are reduced to below the previously stated lower limit of 100 notifications in the year. The measure of completeness of reporting, $\pi$, is shown in the final column of the table.

It can be seen immediately that the values of $\pi$ are higher in the hospitals visited regularly, thus indicating more complete reporting from this group. The values in this group with one exception lie between 80 and 90%. In the "unvisited" group all values of $\pi$ are below 80%, ranging from 77·3% down to 9·6%. The extent to which pathologists contribute notifications also varies widely. In 6 hospitals (Nos. 1, 10, 11, 13, 14 and 18) the contribution was negligible. In other

TABLE II.—*Methods of First Notification for Patients Treated in* 1971 *at* 19 *Principal Hospitals in the MRCR Area*

| | 1 MRO | 2 Path | 3 D.C. | 4 Total | 5 $\pi$ (%)* |
|---|---|---|---|---|---|
| Visited regularly | | | | | |
| 1. | 176 | 3 | 21 | 200 | 89·5 |
| 2. | 226 | 91 | 72 | 389 | 81·5 |
| 3. | 129 | 58 | 31 | 218 | 85·8 |
| 4. | 7 | 35 | 24 | 66 | 63·6 |
| 5. | 103 | 40 | 28 | 171 | 83·6 |
| 6. | 77 | 29 | 15 | 121 | 87·6 |
| 7. | 205 | 71 | 41 | 317 | 87·1 |
| Not visited regularly | | | | | |
| 8. | 52 | 41 | 69 | 162 | 57·4 |
| 9. | 21 | 106 | 82 | 209 | 60·8 |
| 10. | 97 | 5 | 78 | 180 | 56·7 |
| 11. | 66 | 2 | 20 | 88 | 77·3 |
| 12. | 45 | 11 | 20 | 76 | 73·7 |
| 13. | 125 | 0 | 132 | 257 | 48·6 |
| 14. | 40 | 0 | 75 | 115 | 34·8 |
| 15. | 28 | 19 | 58 | 105 | 44·8 |
| 16. | 34 | 19 | 26 | 79 | 67·1 |
| 17. | 8 | 77 | 77 | 162 | 52·5 |
| 18. | 10 | 1 | 103 | 114 | 9·6 |
| 19. | 1 | 82 | 84 | 167 | 49·7 |

* Col 5 = {(Col (1) + Col (2))/Col (4)} × 100.

hospitals (Nos. 4, 9, 17 and 19) the pathologist provided the bulk of the notifications from that hospital.

The efficiency of a disease registration scheme in a large population must always be dependent upon the lines of communication between the registry staff and those who are in a position to provide the first notification. In the case of cancer registration in this country, not based on the HAA scheme, the 2 important sources of information are the hospital MRO and the pathologist. If these sources do not provide the necessary first notifications to the registry, the registration scheme is reduced in essence to a mortality registry, being based solely on the copies of death certificates sent by the Registrar General.

The results in Table II imply that reporting from a hospital is improved by regular visits to the registry. The proximity of the hospitals in the "visited" group to the MRCR (which is in Bebington, Wirral, on the same site as the Clatterbridge Hospital) cannot be an important factor, since some hospitals in the "unvisited" group are even closer (Nos 8,10 and 16). Close ties between the MRCR and the "visited" group of hospitals were built up when the MRCR was housed in Liverpool. However, there is evidence that these ties have to be supported by regular visits, since hospital No. 10 was a hospital which used to be visited up to 1970, yet it is seen from Table II that reporting was low for 1971. Hospital No. 19 was another which had been visited regularly before 1970. The one hospital in the "visited" group with a value of $\pi$ lower than 80% has for local reasons never cooperated fully with the MRCR and in the main relies on the MRCR to make the first approach for any case sheet which is to be reviewed. It appears, from these examples, that regular visits do for the most part improve the MRO's reporting. It is not likely, however, to affect the reporting of pathologists. This is because pathologists report directly to Dr E. McConnell, the registry pathologist, who is well known in the region. The reporting of pathologists is thus a matter of professional agreement between 2 scientists and is likely to be affected only by a change of senior pathologist at the hospital.

Two hospitals which had very few first notifications by the MRO (Nos. 17 and 19), had a level of $\pi$ as high as 50%. It seems likely that this is as high a level as can be achieved without the participation of the MRO in the scheme. On the other hand, Hospital No. 1 had very few first notifications from the pathologist, yet through the reporting by the MRO achieved the highest value of $\pi$ among the 19 hospitals considered.

The measure of completeness of reporting, $\pi$, used here can only provide a rough guide to the true proportion of patients which a hospital *would* report if a first notification were to come only from hospital (including the pathologist) and not from other sources such as death certificates. We refer to this proportion as the "reporting rate" of the hospital. Under certain assumptions (Freedman, 1976) $\pi$ can be shown to be the best estimate available of the reporting rate. The 5 assumptions rigorously stated are as follows.

1. If a patient resident within the MRCR area dies and cancer is mentioned on the death certificate, a copy is sent to the MRO one month after death.
2. If a hospital (MRO or pathologist) reports a case of malignancy to the MRCR, it will do so within 3 months of the anniversary date.
3. For a given hospital, the reporting rate is independent of the patient's disease (anatomical site) and type of treatment.
4. Patients who die some time after being diagnosed in the MRCR area as having cancer, will always have a copy of the death certificate sent to the cancer registry.
5. DCRs only arise when the deceased did not attend hospital in the MRCR area.

It is clear, however, that some of these assumptions will not hold true, especially Assumptions 3, 4 and 5. A full discussion of this problem would be out of place in this paper. Nevertheless, the value of $\pi$ probably comes closer to an assessment of the reporting rate than other methods which have been used and hence, indirectly, closer to an assessment of the level of completeness achieved.

It should be noted that low reporting rates, such as those in the bottom half of Table II, do not automatically imply a large deficiency of all types of registrations in a Cancer Registry. For example, when studying diseases such as lung or stomach cancer where the mortality rate is high, many cases which are not reported by a hospital will be notified to the registry through the death certificate, although the small number of patients who survive to after the date of study, or who moved from the registry area before death, or for whom the death certificate omits mention of cancer, are likely to be lost to registration. Careful consideration should be paid to the reporting rates, however, when assessing the incidence of diseases with a lower mortality rate such as oral cancer, breast cancer, colorectal cancer, cervical cancer, and squamous-cell carcinoma of the skin. This is particularly important when making comparisons of incidence in different areas and times. In conclusion, the results of this study show that within the MRCR area (i) there are wide variations in the level of reporting by hospitals, and (ii) regular visits by the registry staff appear to improve the level of reporting of a hospital. These conclusions may well apply to other Registry areas, and further studies are indicated.

I thank Dr J. A. H. Waterhouse, Dr D. N. Edwards and Dr S. Rubin for kindly reading the draft manuscript and for their helpful comments. I also thank Miss Sheila Murray for typing this paper.

### REFERENCES

ADAMS-JONES, D. & MORLEY-DAVIES, R. B. (1976) *The Welsh Hospital Board Cancer Registry Report*, 1969–73.

ALDERSON, M. R. (1974) Central Government Routine Health Statistics. In *Reviews of United Kingdom Statistical Sources*. Vol. II. London: Heinemann. p. 33.

FREEDMAN, L. S. (1976) Analysis of Treatment Results from Retrospective Cancer Registry Data. In *Computers in Radiotherapy in Europe. Br. Inst. Radio., Special Report*, **13**, Part 2.

GILLIS, C. R. (1969) *Cancer Registration Bureau: 9th Annual Report of the Regional Cancer Committee*. Scotland: Western Regional Hospital Board.

HILL, G. B., HOWITT, L. F. & SOAPER, A. (1972) *Cancer Incidence in Great Britain* 1953–66. Studies on Medical and Population Subjects No 24. London: HMSO.

PAYNE, P. M. (1969) *The South Metropolitan Cancer Registry Annual Report*.

STEWART, M. A. (1973) *Analysis of Malignant Cases Registered* 1969 *&* 1970*: Liverpool Hospital Region, North Wales and Isle of Man*.

WATERHOUSE, J., MUIR, C., CORREA, P. & POWELL, J. (1976) *Cancer Incidence in Five Continents*. Vol. III. Lyon: IARC. Chapter 6. p. 45.